# Historical contingency limits adaptive diversification in a spatially structured environment

**DOI:** 10.1093/evlett/qraf048

**Published:** 2025-12-16

**Authors:** Gillian E Patton, John C Meraz, Michelle Yin, Sarah B Worthan, Sara Williams, Megan G Behringer

**Affiliations:** Department of Biological Sciences, Vanderbilt University, Nashville, United States; Edison Family Center for Genome Sciences & Systems Biology, Washington University School of Medicine, St. Louis, United States; Biodesign Center for Mechanisms of Evolution, Arizona State University, Tempe, United States; Department of Biological Sciences, Vanderbilt University, Nashville, United States; Department of Biological Sciences, Vanderbilt University, Nashville, United States; Evolutionary Studies Initiative, Vanderbilt University, Nashville, United States; Department of Biological Sciences, Vanderbilt University, Nashville, United States; Evolutionary Studies Initiative, Vanderbilt University, Nashville, United States; Department of Biological Sciences, Vanderbilt University, Nashville, United States; Evolutionary Studies Initiative, Vanderbilt University, Nashville, United States; Department of Pathology, Microbiology, and Immunology, Vanderbilt University Medical Center, Nashville, United States

**Keywords:** range expansion, experimental evolution, fitness landscape, evolutionary trap, *Escherichia coli*, biofilms

## Abstract

Understanding how genotype-by-environment (G × E) interactions influence evolutionary trajectories and contribute to historical contingency is key to predicting adaptation. In structured environments, populations often diversify into ecotypes. This specialization depends on ecological opportunity and also hinges on the adaptive landscape, as early beneficial mutations may restrict access to new niches unless alternative trajectories or compensatory mutations arise. Previous studies demonstrated that *Escherichia coli* populations rapidly diversify into two coexisting ecotypes in nutrient-rich, spatially structured environments, mediated by first-step mutations that upregulate type 1 fimbriae, a pilus involved in biofilm formation that enables surface colonization. Here, we investigated how first-step mutations shape evolutionary trajectories by experimentally evolving wild-type and fimbrial-deficient (Δ*fimA*) *E. coli* in structured and unstructured environments. In structured environments, *ΔfimA* initially confers a fitness benefit by eliminating the energetic cost of weak biofilm formation, but ultimately prevents range expansion, constraining adaptation relative to wild-type populations. In unstructured environments, where biofilms provide no advantage, both genotypes evolved similarly, with sequencing revealing parallel early mutational trajectories. Our findings provide one of the first experimental demonstrations that a single, clinically relevant first-step mutation in a non-essential gene can create an evolutionary “dead end,” constraining subsequent diversification. These results highlight the ruggedness of adaptive landscapes in structured environments and show how early beneficial mutations can trap lineages on local fitness peaks, underscoring the role of G × E interactions in shaping the predictability and contingency of evolution.

## Introduction

When faced with a novel environment, organisms must adapt in order to thrive. However, populations with similar genetic backgrounds may follow different adaptive trajectories due to the influence of chance and evolutionary history (Beatty, 2008; Blount et al., 2008; Xie et al., 2021). Here, the term “chance” includes novel mutations and genetic drift, which stochastically shape genetic variation (Mani & Clarke, 1997; Wahl & Krakauer, 2000; Walsh & Lynch, 2018). History is the genetic and developmental background of the evolving population, which can limit the accessibility of different adaptive trajectories (Franke et al., 2011; Futuyma, 2010; Poelwijk et al., 2007; Travisano et al., 1995). Because an early beneficial mutation could render an otherwise more optimal adaptive trajectory inaccessible and change the direction of evolution, a population’s adaptability, or capacity to adapt to a specific environmental condition, is highly contingent upon history and chance (Jerison et al., 2017; Kryazhimskiy et al., 2014; Li et al., 2019; Perfeito et al., 2014; Szamecz et al., 2014).

The repeatability of how natural selection mediates history and chance while navigating the adaptive landscape can be investigated through the experimental evolution of microbial populations (McDonald, 2019). Microbes are an ideal system as their large population sizes and quick generation times allow for the assessment of evolutionary processes over relatively short timescales. A common observation in experimental evolution studies is genetic parallelism between populations that evolved in similar environments, demonstrating that shared selective pressures result in the evolution of genetic similarities (Graves et al., 2017; Le Gac et al., 2013; Ostrowski et al., 2008; Toprak et al., 2011). However, even under extreme selection pressures, such as sub-lethal stress, evolution is not 100% repeatable. One reason is genotypic redundancy, where multiple mutations can produce the same phenotype (Láruson et al., 2020; McDonald et al., 2009; Simões et al., 2019). Genetic incompatibilities can further limit parallelism as two independently beneficial mutations can exhibit sign epistasis, conferring reduced fitness when combined (Kvitek & Sherlock, 2011). Such incompatibilities can constrain populations to very different evolutionary trajectories, which can curtail adaptation if a population becomes stuck on a locally adaptive peak (Maharjan & Ferenci, 2013; Ono et al., 2017). In a complex environment containing multiple spatial niches, a population’s inability to expand its range and inhabit a new niche may constrain its ability to evolve increased fitness (Bridle & Vines, 2007; Polechová & Barton, 2015). Thus, if an early beneficial mutation ultimately limits range expansion, or the ability of a species to expand its distribution and colonize new niches, it may lead to an evolutionary dead end (Agnarsson et al., 2006; Day et al., 2016; Mayrose et al., 2015). To examine how genotype and environment interact to shape adaptability, we will assess how a single mutation can affect adaptation to different culture environments using biofilm formation in *Escherichia coli* as a model.

Biofilms are collaborative interactions of bacterial cells that confer tolerance to stress, antibiotics, and host immunological defenses (Hall-Stoodley et al., 2004). *Escherichia coli*, like many other species of bacteria, forms biofilms in environments where collaboration may be necessary to take advantage of a particular niche or where protective measures are required (Beloin et al., 2008; Hung et al., 2013; Schembri et al., 2003). In addition to defense, biofilms confer many other benefits, including greater access to oxygen at the surface-air interface of a liquid, limitation of competition, and facilitation of cooperation (Armitano et al., 2014; Henriksen et al., 2022; Jefferson, 2004; Oliveira et al., 2015; Xavier & Foster, 2007). In laboratory environments, *E. coli* can form pellicle biofilms at the surface-air interface through adhesion with cell surface appendages called fimbriae (Beloin et al., 2008; Colón-González et al., 2004; Golub & Overton, 2021). The *E. coli* genome contains 14 different operons that encode adhesins, including type 1 fimbriae (*fim*), curli fimbriae (*csg*), antigen 43 (*flu*), and the *E. coli* common pilus (*ecp*) (Hasman et al., 2000; Klemm & Schembri, 2004). Although the genes for the other fimbrial adhesins are intact and functional when genetically engineered to be expressed, *E. coli* K-12 MG1655 only expresses the *fim* and *flu* operons under standard laboratory conditions (Korea et al., 2010). Biofilm formation via the *fim* operon is a phase-variable phenotype whereby two site-specific recombinases, FimB and FimE, control expression by inverting the *fim* promoter (*fimS*) into the “on” or “off” orientation (Dorman & Higgins, 1987; Klemm, 1986). *fimA* is the most highly expressed of the seven genes in the *fim* operon and is essential to type 1 fimbriae formation (Puorger et al., 2011). As a typical type 1 fimbria can contain ~1,000 subunits of FimA, and a fimbriated cell can express 200–500 fimbriae on its surface, expression of the *fim* operon and forming a biofilm represents a substantial energetic commitment, especially when *E. coli* K-12 MG1655 cells are generally weak biofilm formers in standard laboratory conditions and are unable to fully realize the spatial and oxygenation benefits of biofilm formation (Puorger et al., 2011; Reisner et al., 2006).

Despite the steep energetic costs, mutations causing increased biofilm-forming activity through the overexpression of the *fim* operon are a common early adaptive step during the experimental evolution of *E. coli* populations in culture tubes (Behringer et al., 2018, 2022; Ho et al., 2021). In these populations, biofilm formation precedes diversification as *E. coli* evolves to expand its range within the culture tube by colonizing both the highly aerobic surface-air interface and the less aerobic planktonic/bottom-dwelling niche (Behringer et al., 2018). As such, *E. coli* biofilm formation represents a useful model for studying adaptability via eliminating access to a first-step mutation. By deleting *fimA* and thus preventing biofilm formation via the *fim* operon, we can then repeat evolution and survey the adaptive landscape to identify alternative evolutionary trajectories and how these trajectories translate into increased fitness (Jerison et al., 2017; McDonald et al., 2009; Salverda et al., 2011).

Moreover, because the fitness of a particular genetic background is dependent on the environment (Hietpas et al., 2013; Sæther & Engen, 2015; Wadgymar et al., 2024), differences between culture vessels, such as their shape and aeration capabilities, can significantly alter *E. coli*’s physiology and, by extension, its evolution (Worthan et al., 2023). A well-shaken culture flask is essentially a non-structured environment where the medium is more oxygenated and homogenized, while a culture tube is a much more structured environment, where the presence of an oxygen gradient can give rise to the creation of additional distinct niches, simulating some of the complexity of natural environments. If biofilm formation is essential to adaptation in culture tubes, we expect the *E. coli* populations would evolve to activate one of the other 11 cryptic fimbriae operons (Korea et al., 2010). Alternatively, if *E. coli* populations do not activate cryptic fimbriae, either biofilm formation is not essential to adaptation in culture tubes or mutations that enable cryptic fimbriae are not easily accessible. As biofilm formation provides limited benefits in culture flasks due the homogeneity of the environment, we expect minimal differences in adaptability based on a population’s initial biofilm-forming potential (Abberton et al., 2016).

**Figure 1.**
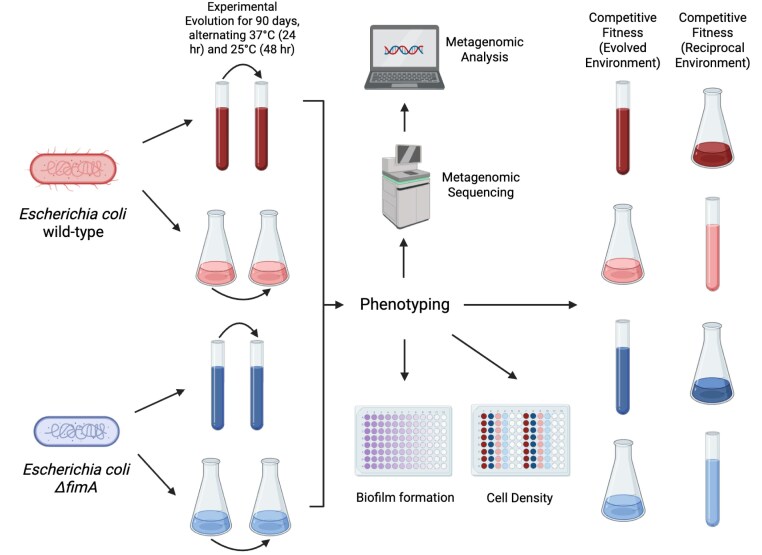
Illustration of experimental methods. We experimentally evolved a total of 24 parallel *E. coli* K-12 MG1655 populations across 4 different treatments: WT-tube WT-flask, *ΔfimA*-tube, and *ΔfimA*-flask. After 91 days of experimental evolution (13 weeks), the evolved populations were compared to their ancestors via biofilm formation assays, competitive fitness assays, and metagenomic analysis.

In this study, we investigate how interactions between genetic background and environment influence adaptability by evolving *E. coli* populations with two different initial genotypes, WT (biofilm-enabled) and Δ*fimA* (biofilm-inhibited), in two distinct environments, culture flasks (no spatial structure) and culture tubes (spatially structured), for 91 days (13 weeks, 215 generations) (Figure 1). Throughout this experiment, we measured changes in culture density and biofilm production. After 91 days of evolution, we assessed changes in fitness suggesting differences in local adaptation, while whole-population metagenomic sequencing reveals genotype-by-environment effects on molecular evolution.

## Results

### Environmental conditions have a distinct effect on biofilm formation and culture density

**Figure 2.**
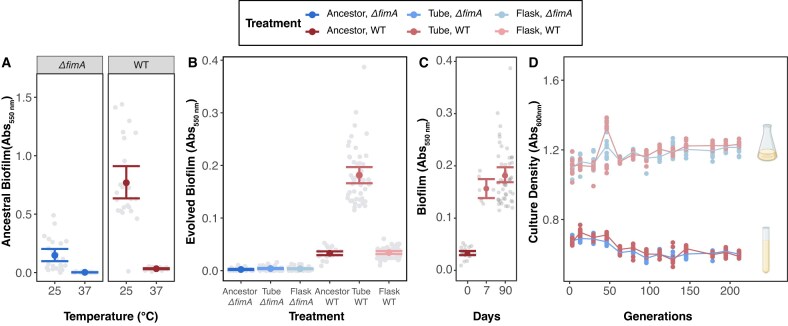
Biofilm production increases only in WT populations experimentally evolved in the tube environment. Biofilm production was assessed following static growth in a 96-well plate. (A) Ancestral capacity for biofilm production after 48 hr at 25°C and 24 hr at 37°C (*n* = 48, 8 replicates across 6 plates). (B) Biofilm production after 91 days of experimental evolution (*n* = 48, 8 replicates across 6 populations per treatment), and (C) biofilm production of WT-tube populations measured after 0, 7, and 91 days of experimental evolution (*n* = 6). (D) Pre-transfer density of cultures assessed every 7 days throughout the evolution experiment (*n* = 6). For panels A–C, light gray circles represent measurements of each population (8 replicates). Colored circles indicate the mean for each treatment, and error bars represent 95% confidence intervals of the mean. For panels B–D, biofilm or culture density was assessed after 24 hr at 37°C (48 hr at 25°C is presented in Figures S1-2). For panel D, colored circles represent the cell density measurements of each population, and colored lines connect the means of each timepoint to visualize trends.

As increased biofilm formation via overexpression of *fimA* is often the first phenotype observed when *E. coli* evolves in a structured culture tube environment (Behringer et al., 2018, 2022; Ho et al., 2021), we replayed evolution to determine how adaptation is affected when evolving populations are unable to form biofilms. We deleted the major subunit of type 1 fimbriae (*ΔfimA*) from the WT experimental ancestor strain to create a biofilm-deficient experimental ancestor and assessed the baseline biofilm production of both genetic backgrounds in a 96-well plate (O’Toole, 2011). Baseline assessment of biofilm production confirmed that the *ΔfimA* mutant did not produce biofilm in either culture condition, while consistent with previous reports, growth for 48 hr at 25°C induces biofilm formation in the WT ancestor (Dorman & Ní Bhriain, 1992; Ingle et al., 2011; Mathlouthi et al., 2018; White-Ziegler & Davis, 2009) (ANOVA with Tukey’s HSD, WT: *P*_37°C vs. 25°C_ = 4.82 × 10^−10^; Figure 2A, Figure S1).

We then initiated 24 experimental populations from the WT and *ΔfimA E. coli* strains. Each population was cultivated via serial-transferred batch culture over 91 days (215 generations) in either a heterogeneous culture tube environment or a more homogeneous culture flask environment (four genetic background/culture condition treatments; six populations each), and after 91 days of experimental evolution, we re-assessed the populations’ ability to form biofilms. Here, we found that all *ΔfimA* populations remained unable to produce biofilms, while WT-flask populations lost their ancestral ability to produce biofilms during 48 hr growth at 25°C (Figure 2B, Figure S1). In contrast, the WT-tube populations gained the ability to form biofilms during 24 hr growth at 37°C (ANOVA with Tukey’s HSD, WT-tube*: P* = 1.78 × 10^−11^), and this increase in biofilm formation could be observed as early as the day 7 timepoint (ANOVA with Tukey’s HSD, *P*_Day 0 vs. 7_ = 4.83 × 10^−5^; *P*_Day 7 vs. 91_ = 0.78; Figure 2C). Given this rapid change in phenotype, we expect the initial increase in biofilm is due to retention of the phase variable expression of the *fim* operon following the first iteration of 48 hr growth at 25°C (Dorman & Ní Bhriain, 1992; Saldaña-Ahuactzi et al., 2022).

As any evolved increases in culture density could skew the ability to detect differences in biofilm formation, we tracked the evolution of culture density weekly throughout the experiment. Although both WT and *ΔfimA* populations evolved changes in culture density, these changes were restricted to the 24 hr/37°C transfers and are unlikely to be a contributing factor to the evolved differences in biofilm formation as populations that evolved in tubes are consistently less dense than populations that evolved in flasks (ANOVA with Tukey’s HSD, *P*_tubes vs. flasks_ = 4.79 × 10^−10^, Figure 2D, Figure S2). This reduced culture density in tubes is consistent with slower growth due to heterogeneous oxygen and resource conditions in culture tubes (Worthan et al., 2023).

### Inability to form biofilms has varying effects on evolved fitness based on environment

**Figure 3.**
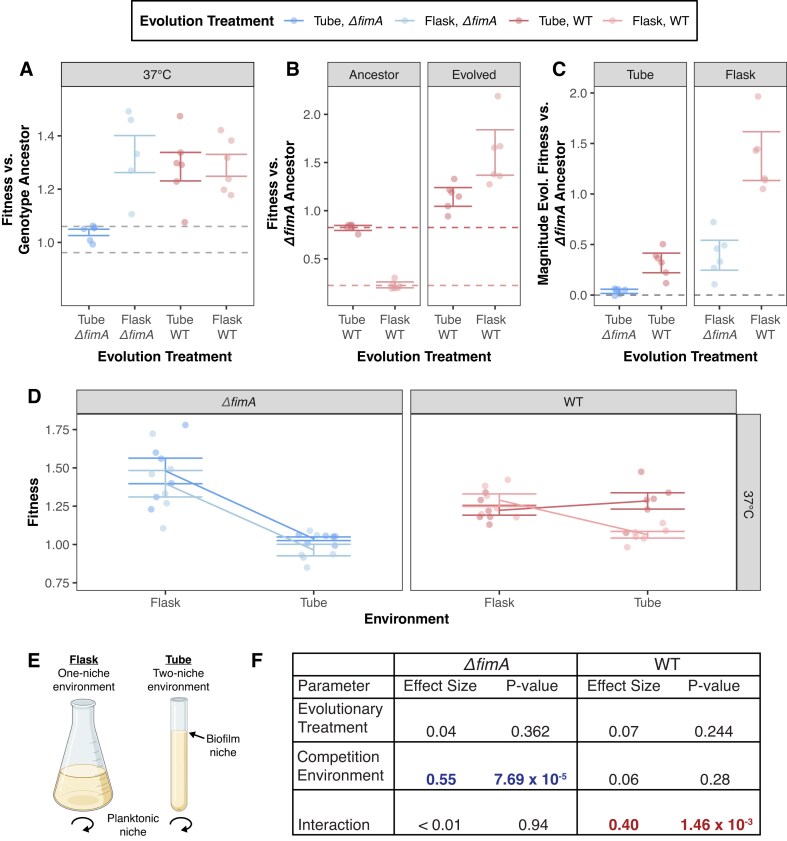
Loss of *fimA* creates an advantage in homogeneous environments and a disadvantage in heterogeneous environments. Fitness is assessed by competitive co-culture in evolved and reciprocal environments. For each treatment, *n* = 6. (A) Fitness of each population in their evolved environment after 91 days relative to the ancestor strain of the same genetic background (e.g., evolved *ΔfimA* tube with *ΔfimA* ancestor). (B) Fitness of ancestral (left) and evolved (right) wild-type populations relative to the *ΔfimA* ancestor assessed by competitive co-culture. (C) Magnitude of the evolved change in fitness relative to the *ΔfimA* ancestor assessed by competitive co-culture. (D) Fitness of each population in their evolved or reciprocal environment relative to the ancestor strain of the same genetic background. Lines illustrate fitness relationships by connecting the mean fitness for each evolved genotype-environment combination. For panels A–D, fitness assays were conducted at 37°C (25°C fitness assays are presented in Figure S3); colored circles indicate fitness values for each population, and error bars represent 95% confidence intervals of the mean. (D) Diagram of the suspected differences in niche space between culture flask and tube environments. (E) Effect size of evolutionary treatment and competitive environment for each genetic background cultured in their evolved or reciprocal environment determined via multiple linear regression. Effect size is calculated as partial eta^2^, and significant parameters are highlighted in blue (*ΔfimA)* or red (WT).

Since we observed a notable increase in biofilm formation from WT-tube populations as early as day 7 that was retained throughout experimental evolution, we suspect that an ability to increase biofilm production is likely an important trait for adaptation in the culture tube environment. To determine how the ability to produce biofilm influences competitive fitness, we co-cultured the evolved populations with their experimental ancestor in their respective evolution conditions (Worthan et al., 2023). Overall, the greatest changes in fitness are observed in the 24 hr/37°C culture conditions, where all evolved populations exhibit increased fitness relative to their ancestor (Figure 3A). Notably, the mean fitness for *ΔfimA-*tube populations is only 1.04, which is within the range of error of the *ΔfimA*-ancestor and quite modest compared to all other evolutionary treatments whose fitness range from 1.29 to 1.40 in 24 hr/37°C conditions. As marginal changes in fitness are observed for *ΔfimA* populations cultivated in tube but not flasks, this suggests that an inability to produce biofilms may only hinder adaptation in the tube environment. Alternatively, fitness changes were modest across all evolved populations in the 48 hr/25°C culture environment with only WT-flask populations exhibiting significantly greater fitness than their ancestor (*P*_WT-flask_ = 0.54, Figure S3). This bias in evolved changes contributing to greater fitness gains in the 24 hr/37°C conditions, instead of both conditions, highlights the specificity of adaptation and suggests that the greatest advantage can be obtained through evolved changes that confer benefits specific to the 24 hr/37°C condition. As such, we focused our following investigations of fitness on these conditions.

Given the modest magnitude of fitness evolution exhibited by *ΔfimA-*tube populations, we considered the possibility that the *ΔfimA* experimental ancestor may have started the evolution experiment closer to the fitness optimum than the WT experimental ancestor. Here, a more limited fitness increase would be expected due to diminishing returns epistasis compared to the contrasting extreme of significant fitness gains by WT-flask populations (Barrick et al., 2009; Chou et al., 2011; Wei & Zhang, 2019; Wünsche et al., 2017). Competition between the two experimental ancestors revealed that the WT ancestor is significantly less fit compared to the *ΔfimA* ancestor in both culture vessel environments (one-sample t-test, *P*_tube_ = 8.4 × 10^−5^, *P*_flask_ = 1.1 × 10^−7^, Figure 3B), particularly in flasks, where the WT ancestor was essentially undetectable after 24 hr of co-culture with the *ΔfimA* ancestor. However, competing the evolved WT populations against the *ΔfimA* ancestor revealed that WT populations surpassed the fitness of the *ΔfimA* ancestor by the end of the evolution experiment (one-sample t-test, *P*_tube_ = 0.047, *P*_flask_ = 0.0079) and exhibited a greater magnitude of fitness increase relative to the *ΔfimA* ancestor than the evolved *ΔfimA* populations (one-sample t-test, *P*_WTtube_ = 3.2 × 10^−3^, *P*_WTflask_ = 2.7 × 10^−4^, Figure 3C). These results suggest that while the *ΔfimA* ancestor may have begun experimental evolution with an initial fitness advantage, there is still considerable room for fitness improvement, and *ΔfimA-tube* populations may be stuck near a local fitness peak.

Finally, to determine if these evolved fitness outcomes were generalizable across environments, we assessed for signatures of local adaptation by repeating the competitive fitness assays in reciprocal environments (ex. assessed culture tube-evolved populations in flask environments). All populations evolved under flask conditions exhibit clear evidence of local adaptation, performing best in their evolved conditions (flasks) with negligible fitness changes in the alternative environment (tubes). (ANOVA with Tukey’s HSD, *P*_Evolutionary treatment: Competition conditions_ = 1.42 × 10^−9^, Figures 3D–F). In contrast, *ΔfimA*-tube populations exhibited greater fitness in flasks than in tubes, whereas WT-tube populations exhibited increased fitness in both environments. This pattern suggests that adaptation in the tube environment is contingent upon the ability to form biofilms and to undergo range expansion. The perceived lack of increased fitness for *ΔfimA*-tube populations in their evolved, spatially structured environment may reflect a shift toward a planktonic specialist strategy, that is revealed only in the unstructured flask environment. Conversely, the WT-tube populations appear to have evolved a range generalist strategy, improving their performance across both the biofilm and planktonic niches within the tube environment (Figure 3E) (von Meijenfeldt et al., 2023).

### Mutational parallelism illustrates genotype-by-environment effects on evolutionary trajectories

**Figure 4.**
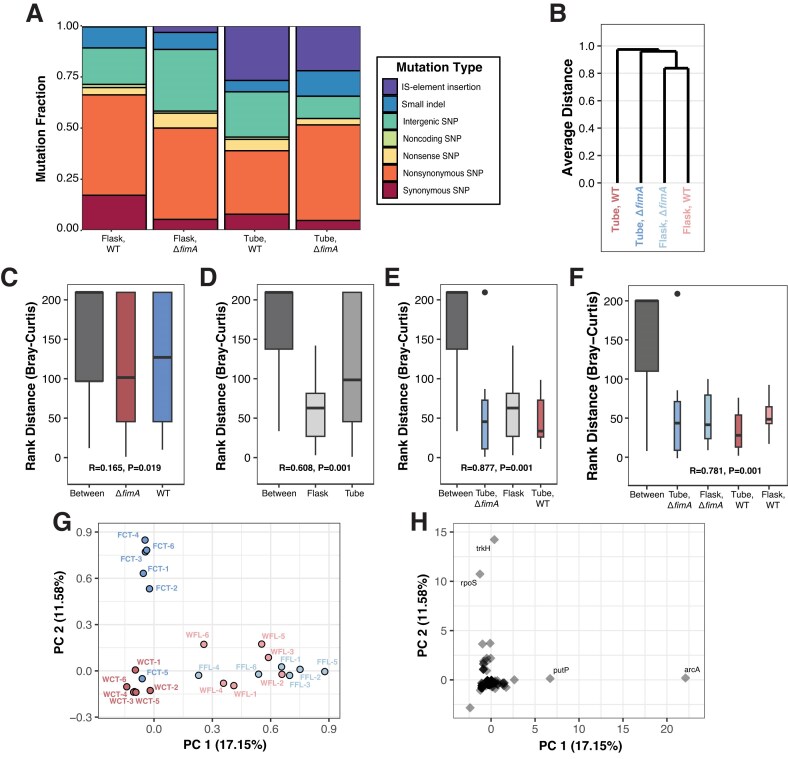
Mutational spectrum and parallelism are influenced by genotype-by-environment interactions. Metagenomic sequencing of evolved populations allowed the assessment of mutation spectrum and mutational parallelism. (A) Breakdown of mutations contributing to evolution in each genotype-environment combination grouped by mutation type. Colors indicate different mutation types. (B) Average mean mutational distance between each genotype-environment combination computed based on Bray–Curtis dissimilarity. Rank distance based on Bray–Curtis dissimilarity comparing (C) genetic background, (D) evolution environment, (E) evolution environment with tube populations split by genetic background, and (F) all populations split by environment and genetic background. For rank distance, lower values indicate greater similarity between grouped populations, and best similarity fits are indicated by higher R values. (G) Principal component plot of evolved populations and (H) loci with nonsynonymous mutations. Principal component analysis was based on number of nonsynonymous mutations at each locus for each population. Panels G and H are from the same analysis and are plotted on the same coordinate space. Three letter designations describe the different genotype/environment combinations and the population number, FCT: Δ*fim A*, tubes; FFL: Δ*fim A*, flasks; WCT: WT, tubes; WFL: WT, flasks. For panels B—G, colors indicate each genotype-environment combination.

Characterization of adaptive trajectories and determination of how genotype-by-environment interactions influence the effects of first-step mutations require that we examine changes in the genome. To assess evolution on the molecular level and look for genetic patterns of positive selection, such as evidence of mutational parallelism, we performed metagenomic sequencing on the evolved populations at the 91-day timepoint (Good et al., 2017). We also sequenced ancestor populations to remove any shared variation that could have been introduced when constructing the ancestral strains. After quality filtering to remove ancestral, low-quality, and low-frequency (<10%) polymorphisms, we identified 505 total mutations across all evolved populations (Table 1, Table S1). For all populations, nonsynonymous SNPs represented the largest proportion of mutations, followed by intergenic SNPs, suggesting that the majority of mutations that increased to a frequency of > 10% likely resulted in an alteration of a gene’s function or expression as opposed to loss of function. Consistent with previous observations in digital organisms, which demonstrated that rugged fitness landscapes were associated with slower genomic evolutionary rates (Clune et al., 2008), populations cultivated in tubes—expected to represent a relatively rugged fitness landscape compared to flasks—exhibited a lesser magnitude of genomic evolution than flask populations. Another striking difference is the mutation spectrum, where IS-mobile elements are a greater contributor to evolution in tube populations compared to flask populations (Figure 4A).

**Table 1.** Mutations per treatment.

	Background	Environment	Single nucleotide polymorphisms (SNPs)					Structural variants (SVs)			
			Nonsense	Nonsynonymous	Synonymous	Noncoding	Intergenic	Small indel	Small insertion	Small deletion	IS-element insertion
Total mutations	WT	Flask	9	146	43	4	57	32	10	22	1
	*ΔfimA*	Flask	9	54	6	1	37	11	1	10	3
	WT	Tube	7	31	7	1	32	5	3	2	27
	*ΔfimA*	Tube	2	34	3	0	20	8	3	5	14
Average mutations (std. err.)	WT	Flask	1.5 (0.5)	24.33 (2.2)	7.17 (1.38)	0.67 (0.49)	9.5 (0.67)	5.33 (1.28)	1.67 (0.49)	3.67 (1.02)	0.17 (0.17)
	*ΔfimA*	Flask	1.5 (0.34)	9 (1.29)	1 (0.52)	0.17 (0.17)	6.17 (1.8)	1.83 (0.6)	0.17 (0.17)	1.67 (0.67)	0.5 (0.22)
	WT	Tube	1.17 (0.4)	5.17 (1.22)	1.17 (0.79)	0.17 (0.17)	5.33 (0.99)	0.83 (0.31)	0.5 (0.22)	0.33 (0.21)	4.5 (0.56)
	*ΔfimA*	Tube	0.33 (0.21)	5.67 (0.71)	0.5 (0.22)	0 (0)	3.33 (0.42)	1.33 (0.21)	0.5 (0.22)	0.83 (0.31)	2.33 (0.8)

Considering these differences in the mutational spectrum, we also investigated gene-level mutational parallelism focusing on nonsynonymous mutations. To identify evidence of mutational parallelism, we first calculated average mean distance (Figure 4B) using the Bray–Curtis metric before conducting an analysis of similarity test (ANOSIM) to characterize the similarity of evolutionary trajectories within and between treatment groups (Figures 4C–F) (Bardhan et al., 2024; Behringer et al., 2022; Turner et al., 2018). This analysis revealed that parallelism is best explained when flask populations are treated as a single group and *ΔfimA*-tube and WT-tube populations are each treated as their own groups (ANOSIM: *R* = 0.877, *p* = .001, Figure 4E), suggesting that *ΔfimA*-flask and WT-flask populations share similar gene-level mutational profiles, but *ΔfimA*-tube and WT-tube populations exhibit distinct profiles. To investigate this similarity further, we conducted a principal component analysis using the gene-level nonsynonymous mutation matrix and found a similar pattern where the individual flask populations cluster together while the tube populations cluster based on initial genotype (Figure 4G). This observed clustering of flask populations is consistent with directional selection on these populations and our hypothesis that early evolution under flask environmental conditions represents a relatively simple adaptive landscape.

**Figure 5.**
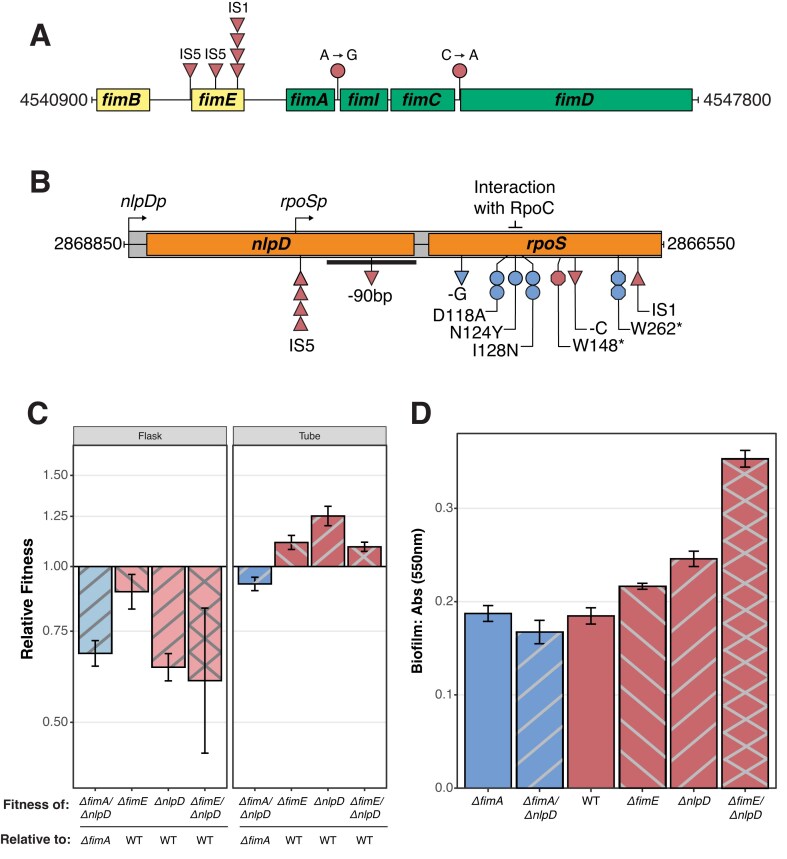
Mutations disrupting *nlpD* are beneficial in tube environments and contribute to biofilm formation. (A) Genic region of the *fim* operon and the (B) *nlpD* and *rpoS* operon shown, highlighting mutations observed in this experiment indicated below the labeled genes. Shapes indicate mutation types: insertions, upright triangles; deletions, upside-down triangles; SNPs, circles; nonsense mutations, octagons; colors indicate evolutionary treatment: red, WT-tube; blue, *ΔfimA*-tube. Notably, a *rpoS* promoter (*rpoSp*) embedded within the *nlpD* locus lies upstream of the insertion sites for the IS elements observed in our evolved populations. (C) Competitive co-culture reveals that *ΔnlpD* confers a fitness advantage in the tube environment, but not in the flask environment (*n* = 3 for genotype per environment). (D) Biofilm formation assays of *ΔnlpD* mutants, both alone and in combination with *ΔfimE* and *ΔfimA*, reveal an additive effect between the *ΔfimE* and *ΔnlpD* mutations (*n* = 14 replicates per genotype). Error bars represent 95% confidence intervals of the mean.

Additionally, by plotting the genes we can also gain insight into the characteristic mutations of each evolutionary treatment (Figure 4H). For instance, flask populations are associated with nonsynonymous mutations in *arcA*, a member of the ArcAB two-component system that regulates the switch from aerobic to anaerobic respiration (Iuchi & Lin, 1995). WT-flask and *ΔfimA-*flask populations both contain 12 SNPs in *arcA*, with the majority of *arcA* mutations affecting the response regulator receiver domain (Georgellis et al., 1997; Toro-Roman et al., 2005) (Figure S4). Alternatively, *ΔfimA-*tube populations are associated with nonsynonymous mutations in *trkH* with 7 SNPs in this gene that encodes a K^+^/H^+^ symporter (Schlösser et al., 1995). These SNPs primarily affect residues within the unresolved cytoplasmic loop of the TrkH protein (Cao et al., 2011). In addition to mutations in *trkH, ΔfimA-*tube populations also have 7 SNPs and a small insertion within *rpoS*, with five of these SNPs affecting residues that interface between the RpoS general stress sigma factor and the core RNA polymerase (Cavaliere et al., 2015; Gao et al., 2018; Monteil et al., 2010; Tanaka et al., 1993) (Figure 5A). Finally, while no true characteristic SNPs were identified via PCA for the WT-tube populations, two genes stood out in the genomic data as key targets of IS-element insertions: *fimE* (*fimA* regulating recombinase, IS-1 insertions) and *nlpD* (divisome associated factor, IS-5 insertions), consistent with the original evolution experiment that motivated this study (Behringer et al., 2018; Ichikawa et al., 1994; Klemm, 1986; Lange & Hengge-Aronis, 1994).

### Insertions in *nlpD* are uniquely beneficial in WT backgrounds

As we observe mutations disrupting *fimE* and *nlpD* only in the WT-tube populations and not in the *ΔfimA-*tube populations, these distinct mutations are key candidates for historically contingent adaptive trajectories in the tube environment (Figure 5A, B, Supplemental Table S1). Interestingly, not only are *nlpD* and *rpoS* encoded in the same operon, but the coding region for *nlpD* also contains the *rpoS* promoter—located just upstream of the IS-5 insertions disrupting *nlpD* (Lange & Hengge-Aronis, 1994) (Figure 5B). As such, WT-tube populations may be taking a “two-birds-one-stone” approach as disruptions of *nlpD* will also disrupt *rpoS* expression (Tsunoi et al., 2021). Thus, we expect that ruggedness in the fitness landscape may explain why *ΔfimA-*tube populations evolve mutations that directly disrupt *rpoS* while WT-tube populations instead evolve mutations in *nlpD* (Diaz-Colunga et al., 2023; Kvitek & Sherlock, 2011; Weinreich et al., 2005).

To test this, we constructed *nlpD* and *fimE* deletions, individually and in tandem, in the ancestral WT background, as well as an *nlpD* deletion in the ancestral *ΔfimA* background. Competing these mutants against the WT and *ΔfimA* ancestors in the tube and flask environments revealed that deletions of *nlpD* were only beneficial in the WT-tube conditions (Figure 5C). Addition of *ΔnlpD to* a *ΔfimE* background has a neutral effect on fitness but significantly increases biofilm production (Figure 5C, D) (pairwise t-test, *P_ΔfimE_*_vs._  *_ΔfimE/ΔnlpD_* < 2.2 × 10^−16^; *P_ΔnlpD_*_vs._  *_ΔfimE/ΔnlpD_* = 6.4 × 10^−12^), confirming the contribution of these mutations to enabling range expansion. The large phenotypic effect but minor fitness effect of *ΔnlpD* on a *ΔfimE* background is consistent with pleiotropy leading to sign-epistasis, as disrupting *nlpD* affects both its own function and *rpoS* expression. These results demonstrate an instance of non-linearity between phenotype and fitness (Sackman & Rokyta, 2018), and highlight how genotype-by-environment interactions influence evolutionary trajectories.

## Discussion

Here, we investigate the importance of genotype-by-environment interactions in determining the direction of evolution. Our study provides one of the first experimental demonstrations that a single, clinically relevant first-step mutation in a non-essential gene can create an evolutionary “dead end” by preventing subsequent adaptive diversification. Permissive mutations and those that increase evolvability have been explored experimentally (Barrick et al., 2010; Blount et al., 2008; Cummins et al., 2021; Gifford et al., 2018; Wagner, 2023; Woods et al., 2011) and more recently, theoretically (Ferrare & Good, 2024; Kumawat et al., 2025). However, evolutionary dead-ends and mutations that limit evolvability are of emerging interest, particularly because of their clinical relevance to cancer (Acar et al., 2020; Black & McGranahan, 2021) and pathogen evolution (Lukačišinová et al., 2020). Recently, Sánchez-Maroto et al., demonstrated that a P91Q substitution in ribosomal protein S12 (*rpsL*) confers resistance to streptomycin but impedes subsequent resistance evolution to newer aminoglycoside antibiotics (Sánchez-Maroto et al., 2025). In contrast, our study focuses on the *fim* operon encoding type 1 fimbriae, critical adhesins that promote biofilm formation and the establishment of intracellular bacterial communities during urinary tract infection (Sivick & Mobley, 2010). We demonstrate that disruption of *fimA*, while initially beneficial, traps *E. coli* populations on a constrained adaptive landscape where they fail to activate alternative cryptic fimbriae to compensate. Unlike first-step mutations in essential genes that underlie antimicrobial resistance, this work illustrates how loss-of-function in a virulence factor can act as evolutionary trap, offering a potential strategy for steering pathogen evolution.

As a first-step mutation, transposition of IS-1 into the *fimE* locus increases biofilm formation via the overexpression of the *fim* operon, enabling range expansion and colonization of the surface-air interface within the culture tube environment. However, an alternative first-step disruption of *fimA* is also initially beneficial as it reduces the energetic burden of baseline fimbrial expression, which has been described in other experimental evolution studies conducted in flasks (Tenaillon et al., 2016), under starvation conditions (Yun et al., 2024), and under high osmolarity (Winkler et al., 2014). To assess how first-step mutations can affect evolvability, we replayed evolution for 91 days. We observed that the initial benefits of disrupting type 1 fimbriae are ultimately deleterious, hindering adaptation to the structured environment by prohibiting range expansion. These effects are environmentally dependent, as deletion of *fimA* did not appear to affect adaptability in flasks, where biofilm formation is not advantageous, and WT populations ultimately evolve to repress biofilm formation. On the genomic level, we also observe genotype-by-environment effects on mutational parallelism as populations evolved in flasks exhibit more similar molecular evolution. In contrast, the molecular adaptive trajectories of tube populations are distinct between genetic backgrounds, as they both disrupt *rpoS* but through mutationally different means.

Prior research studies focused on first-step mutations have reported that these mutations often have large effects through adaptive loss of function (Klim et al., 2024; Murray, 2020), widespread alteration of gene regulation (Cohen & Hershberg, 2022; Ho & Zhang, 2018; Rodríguez-Verdugo et al., 2016), and sign-epistasis (Aggeli et al., 2021; Johnson & Desai, 2022; Li et al., 2019). The effect of adaptive loss of function is most apparent in WT-tube populations where transpositions of IS-elements disrupting *fimE* and *nlpD* are the most common first and second-step mutations, sweeping to average frequencies of 0.813 and 0.706, respectively. These high frequency alleles are associated with biofilm formation, indicating that the highest population density is at the range margin, or surface-air interface biofilms, and that there is a greater initial fitness advantage associated with range expansion. As previously observed, biofilm forming clones containing IS-insertions in *fimE* and/or *nlpD* regularly exhibit slower diauxic growth (Behringer et al., 2018). Thus, it’s unlikely that this adaptive gene loss is associated with other cellular advantages, such as minimizing energetic waste.

Theory of r-K dynamics in range expansion predicts K-selection (higher density and slower growth rates) in the range core, and r-selection (lower density and higher growth rates) in the range margin (Burton et al., 2010). However, our study demonstrates the reverse to be true, and other experimental evolution studies have similarly reported evidence of K-selection in range margins (Fronhofer & Altermatt, 2015). For example, Fronhofer and Altermatt observe high densities in *Tetrahymena population* range margins, which they attribute to dispersal-foraging trade-offs as dispersal reduces competition (Fronhofer & Altermatt, 2015). The inability to range expand essentially confines all individuals in *ΔfimA*-tube populations to the range core. Thus, the observation of *ΔfimA*-tube populations ultimately exhibiting higher fitness in flask environments is consistent with increased selection on foraging, as foraging efficiency and growth rate are expected to be greater contributors to competitive fitness in more homogeneous and unstructured conditions (Trevail et al., 2019). This is further supported by the observation that genes with a role in resource uptake and utilization are enriched for higher frequency mutations in *ΔfimA*-tube populations, such as nonsynonymous mutations in *trkH* (K^+^: H^+^ symporter), and disruptive structural variants in the global regulators *cytR* (cytidine regulator) and *rpoS* (general stress response sigma factor). RpoS in particular has a major role in regulating growth and foraging as loss of *rpoS* decreases stress resistance but increases growth and resource utilization (Ferenci, 2005), while CytR represses genes involved in nucleoside uptake and scavenging (Watve et al., 2015). Interestingly, *nlpD*, which acquires IS-element insertions in all WT-tube populations, is located upstream of *rpoS* on the same operon and contains a dedicated *rpoS* promoter within its sequence (Farr et al., 2025; Lange & Hengge-Aronis, 1994; Tsunoi et al., 2021). Thus, disruptions of *nlpD* serve a dual role as they contribute to biofilm formation (Figure 5C), but also downregulate expression of *rpoS*, resulting in broad-scale alterations of gene expression. Other studies have seen historical contingency result in divergent molecular but convergent phenotypic evolution (Blount et al., 2018; McDonald et al., 2009). As such, the divergent molecular but convergent phenotypic evolution of mutations disrupting *nlpD* in WT-tube populations versus mutations disrupting *rpoS* in *ΔfimA*-tube populations further illustrates how historical contingency influences the navigation of the genotype x phenotype landscape.

In addition to the fluctuating conditions used in our studies (Behringer et al., 2018), mutations in *nlpD* and *fimE* were similarly observed during experimental evolution in tubes under constant 37°C temperature (Behringer et al., 2022; Ho et al., 2021). Here, out of 16 populations, all evolved mutations disrupting *fimE* and 8 evolved mutations disrupting *nlpD*. As these studies were independently conducted and initiated within a span of ~5 years with slightly different conditions, this serves an excellent example of how selection can promote early parallel evolution. While this is especially true of mutations mediated by IS-element insertions and small indels, as these events are enriched within specific genomic locations, we also observe a number of mutations that are parallel on the nucleotide level.

Although mutational parallelism at the level of nucleotide position is thought to be rare, there are unique instances when we expect to observe this type of parallelism: (1) stressful conditions with very strong selection: antibiotic stress (Toprak et al., 2011), heat stress (Rodríguez-Verdugo et al., 2014; Tenaillon et al., 2012), starvation stress (Behringer et al., 2022, 2024; Zion et al., 2024), and pH stress (Hamdallah et al., 2018; Worthan et al., 2024); and (2) early polymorphic parallelism due to clonal interference (Good et al., 2017). This is best exemplified in the LTEE where 312 mutations are detected in 2 or more populations at the same nucleotide position (129 nucleotide positions) within the first 2,000 generations (Table S2, Figure S5A). Of these, only 4 mutations ultimately sweep to fixation, representing 1 nucleotide position (*pykF* A301S: Ara + 1, Ara + 5, Ara-5; *pykF* A301T: Ara-6). Similarly, in this study, we observe 97 mutations that occur in 2 or more populations at the same nucleotide position (36 nucleotide positions) (Figure S5B). Of these, 54 mutations occur across multiple genotype x environment combinations (19 nucleotide positions), and only 1 has swept to fixation (*arcA*, F105L, WT-flask—population 1). As such, by sequencing early, we can detect early polymorphic parallelism before it is ultimately erased by the fixation of a singular allele.

Lastly, one surprising outcome of this study is that despite the evolutionary disadvantage, deletion of *fimA* is not rapidly compensated by expression of cryptic fimbriae in the tube environment. In *E. coli*, there are eleven cryptic fimbriae that are similar to the type 1 fimbriae encoded by the fim operon, but are not normally expressed by *E. coli* K-12 under laboratory conditions (Korea et al., 2010). Prior studies have demonstrated that at least six of these cryptic fimbriae encode functional adhesins when artificially expressed (Korea et al., 2010). In our study, *ΔfimA*-tube populations were unable to evolve expression of cryptic fimbriae, which are typically repressed by H-NS, a pleiotropic histone-like nucleoid-binding protein and global repressor that regulates more than 5% of the *E. coli* genomes (Hommais et al., 2001; Olsen & Klemm, 1994; White-Ziegler & Davis, 2009). As such, the ability to evolve expression of cryptic fimbriae may be under extreme genetic constraint as alteration of H-NS may result in severe unintended consequences of spurious gene expression or defects in chromosome organization. Because of this, deletion of *fimA* may be useful for studies using chemostats, which are often complicated by biofilm formation (Bryers, 1984), or FimA may prove to be a useful non-lethal target for therapeutic development (Wu et al., 2024). Ultimately, our study demonstrates how a seemingly beneficial first-step mutation can act as an evolutionary trap, stranding a population on a local adaptive peak.

## Materials and methods

### Strain construction and experimental evolution

To follow up on evolved outcomes observed in Behringer et al., 2018 (Behringer et al., 2018), we replayed evolution for 91 days (215 generations) to determine the effects of genetic background and environmental heterogeneity on *E. coli* adaptation. Here, we experimentally evolved a total of 24 parallel populations of *E. coli* K-12 strain MG1655 that were evenly split across 4 different treatments: WT-tube, WT-flask, *ΔfimA*-tube, and *ΔfimA*-flask. All “WT” *E. coli* populations are descendants of PFM2, a prototrophic derivative of *E. coli* K-12 strain MG1655 (MG1655, *rph*+) (Lee et al., 2012). All *ΔfimA* cultures were constructed on the PFM2 background via P1 transduction by transferring the *fimA*782(del):: *kan* cassette from JW4277-1 (Baba et al., 2006) into PFM2 and disrupting the *fimA* gene. Additionally, to allow for the direct comparison of evolved fitness effects and easily screen for cross contamination, all WT-flask and *ΔfimA*-tube populations also contained a deletion of the *araBAD* operon (Δ*araBAD*567). Disruption of *araBAD* is a known neutral marker commonly used in microbial experimental evolution, which allows strains to be colormetrically differentiated on Tetrazolium/Arabinose agar (TA) (Levin et al., 1977).

To conduct experimental evolution, transfers were conducted as previously described alternating 48 hr of growth at 25°C and 24 hr of growth at 37°C, shaking at 180 rpm (Behringer et al., 2018) (Figure 1). In nature, *E. coli* exhibit a biphasic lifestyle cycling between host-associated and open environments; alternating culture between these temperatures allows us to assess evolution under these ecologically relevant fluctuations. Since *E. coli* growth is approximately twice as fast at 37°C as it is at 25°C (6–8 h to stationary phase at 37°C; 12–14 h to stationary phase at 25°C), alternating transfers between 48 hr of growth at 25°C and 24 hr of growth at 37°C maintains that the time spent in each growth phase is proportional between temperature conditions. Populations were each initiated by inoculating an independent isolated colony from its respective ancestral background into 10 ml of LB broth. Each subsequent transfer consisted of a 1:10 dilution where 1 ml of well-mixed evolving culture was inoculated into 9 ml of fresh LB broth, resulting in ~3.3 generations per transfer.

Populations evolving to culture tube treatments were cultivated in 16 × 100 mm glass culture tubes (VWR, Cat. 47729-576), while populations evolving to flask treatments were cultivated in 50 ml glass culture Erlenmeyer flasks (Pyrex, Cat. 4980). The contrasting vessel geometries were deliberately chosen to manipulate spatial structure: culture tubes promote biofilm formation along the air–liquid interface, whereas Erlenmeyer flasks discourage biofilm development and promote culture homogenization. This design allowed all populations to be incubated under identical environmental conditions, with vessel geometry serving as the sole variable influencing the opportunity for biofilm formation. Every week following a 24 hr/37°C incubation, populations were screened for contamination by streaking 1 μl of culture on TA and McConkey agar plates. To maintain a rich historical record of evolution, each week following the succeeding 48 hr/25°C incubation 1 ml of well-mixed pre-transfer culture was collected and mixed with 40% glycerol for storage at −80°C. Following the 91-day time point, additional 1 ml aliquots were collected for DNA isolation and metagenomic sequencing. Here, the spent media was removed via centrifugation (10,000 × *g* for 2 m), and the culture was flash frozen with liquid nitrogen before storing at −80°C.

To assess phenotypes of mutations identified in this work, we generated *ΔnlpD* and *ΔfimE* mutants in multiple genetic backgrounds. The *ΔnlpD* single mutant strain was generated by moving the *nlpD*747(del):: *kan* cassette from the Keio collection strain JW2712 to PFM2 (WT) using P1 transduction. Similarly, the *ΔfimE* single mutant strain was created by moving the *fimE*781(del):: *kan* cassette from the Keio collection strain JW4276 to PFM2 (WT) using P1 transduction. To produce the *ΔfimE/ΔnlpD* and *ΔfimA/ΔnlpD* double mutant strains, the kanamycin resistance cassette was first excised from the *ΔfimE* and *ΔfimA* single mutant strains. This was accomplished by expressing the FLP recombinase from the helper plasmid pCP20, which resulted in the removal of the kanamycin resistance cassette leaving behind the FRT scar site (Datsenko & Wanner, 2000). Subsequently, the *nlpD*747(del):: *kan* cassette from JW2712 was introduced into the resulting *ΔfimE* and *ΔfimA* strains through P1 transduction, resulting in the *ΔfimE/ΔnlpD* and *ΔfimA/ΔnlpD* strains. All mutants were verified via PCR followed by Sanger sequencing.

### Measurement of growth phenotypes and quantification of biofilm formation

Pre-transfer cell density was measured weekly before a 24 hr/37°C transfer and a 48 hr/25°C transfer over the course of experimental evolution by adding 150 μl of well-mixed culture to a 96-well plate (Corning/Falcon, Cat. 351172) and then recording absorbance at 600 nm in an Epoch2 Microplate Spectrophotometer (Biotek). Cell density measurements for each evolving population were taken in duplicate at each timepoint. To quantify evolved differences in the ability to form biofilms, we used a microtiter plate biofilm assay (O’Toole, 2011). For biofilm formation at 25°C, 15 μl of 25°C/48 hr culture was inoculated into 96-well plates (Corning/Falcon, Cat. 351172) containing 150 μl of LB broth. Inoculated 96-well plates were incubated for 48 hr at 25°C before removing the spent media containing unattached cells and washing the plate three times with 1× PBS. Plates were then stained with 200 μl of 0.1% crystal violet for 10 m before washing again with 1× PBS and left to dry overnight. Crystal violet bound to biofilms was then solubilized with 200 μl 30% acetate for 15 m and transferred to a fresh 96-well plate. Absorbance was quantified using an Epoch2 Microplate Spectrophotometer at 550 nm. To compare biofilm formation between 48 hr/25°C treatments and 37°C/24 hr treatments, this process was repeated with incubation temperatures adjusted to 37°C and incubation times adjusted to 24 hr.

Each evolved population/timepoint combination was measured for a total of 8 replicates, and each 96-well plate assay contained 8 replicates of the WT ancestor (PFM2) and 8 blank wells containing only media to serve as a control. To remove background, the median absorbance of each blank was subtracted from all wells containing cells; any samples with negative absorbances at this point were fixed to an OD of 0.00001. To correct for batch effects between plates due to the preparation of crystal violet, we calculated the median value of the WT ancestor on each plate and normalized WT ancestor values by dividing by the median of all plates. This normalization factor (median plate x ancestor/median ancestor across all plates) was then applied to all samples. Lastly, to determine if differences in biofilm formation could be attributed to evolved differences in cell density, we checked for a changing trend in biofilm measurements after normalizing by dividing by evolved cell density for the relevant time point.

### Estimation of relative fitness

We assessed relative fitness for each 91-day evolved population in comparison to its ancestor (Worthan et al., 2023). Fitness was measured in both the evolved and the reciprocal environments to assess the degree of local adaptation. Ancestor strains were revived from frozen stock on LB agar, while evolved populations were revived in LB broth and incubated at 37°C for 24 hr.

Before competition, cultures were preconditioned in the respective competition environment (tube or flask) overnight at 37°C for 24 hr. After preconditioning, the optical density of these cultures was measured using an Epoch2 Microplate Spectrophotometer at 600 nm and used to calculate the cell density in the overnight cultures (Equation 1) to equalize the concentration of evolved and ancestor cells in 10 ml LB broth.


(1) \begin{eqnarray*}
{\mathrm{Volume}}_{\mathrm{Evolved}} = \frac{{\mathrm{OD}}_{\mathrm{Evolved}} \times {\mathrm{OD}}_{\mathrm{Ancestor}}} {{\mathrm{Volume}}_{\mathrm{Ancestor}}}.
\end{eqnarray*}

Competition tubes and flasks were incubated at 37°C for 24 hr or 25°C for 48 hr. Initial colony forming units (CFU) of each competing genotype were measured at 0 hr and final CFU counts were taken at 24 hr (37°C) or 48 hr (25°C). These initial and final cell counts were used to determine the relative fitness (*W*) of each strain (Lenski et al., 1991) (Equation 2).


(2) \begin{eqnarray*}
W = \frac{{{\mathrm{ln}}\left( {\frac{{{\mathrm{Evolve}}{{{\mathrm{d}}}_{{\mathrm{Final}}}}}}{{{\mathrm{Evolve}}{{{\mathrm{d}}}_{{\mathrm{Initial}}}}}}} \right)}}{{{\mathrm{ln}}\left( {\frac{{{\mathrm{Ancesto}}{{{\mathrm{r}}}_{{\mathrm{Final}}}}}}{{{\mathrm{Ancesto}}{{{\mathrm{r}}}_{{\mathrm{Initial}}}}}}} \right)}}
.\end{eqnarray*}

To determine the proportion of variance in relative fitness estimates associated with evolutionary treatment, culture conditions, or the interaction term, we performed a type 3 ANOVA using the lm function from the stats package on the following model: Fitness ~ Evolutionary Treatment * Competition Environment. Partial eta squared to assess Effect Size was calculated using the partial_eta_squared function from the rstatix package.

### DNA extraction and mutation calling

DNA was extracted from the previously frozen copies of each line using the DNeasy UltraClean Microbial Kit (Qiagen). Library preparation and metagenomic sequencing were performed at the Vanderbilt University Medical Center. The samples were normalized to target 100 ng of total input for each sample. Libraries were prepared using the Twist Biosciences kit (Twist, 104207), assessed using the Agilent Bioanalyzer, and quantified with the KAPA Library Quantification Kit (Kapa, KK4873) and the QuantStudio 12K instrument. Prepared libraries were pooled in equimolar ratios, and 150 bp paired-end sequencing was performed on the NovaSeq 6000 platform targeting 2M reads per library.

Resulting reads were processed using fastp v.0.23.1 to remove residual adapters and trim low-quality sequences (Chen, 2023). After this quality control process, we mapped population-level metagenomic sequencing reads to the reference genome of *E. coli* K-12 substr. MG1655 (NC_000913.3) with the Breseq v.0.35.0 pipeline, calling mutations and their frequencies with the predict-polymorphisms parameter setting (Deatherage & Barrick, 2014). Before statistical analysis, mutations present in each ancestor population were filtered out of the corresponding evolved populations, as well as low quality and low-frequency polymorphisms (<10%). We chose 10% as this was the cutoff where we minimize calling artifacts from library preparation. The total number of polymorphisms identified in the ancestors were WT, flask: 38; WT, tube: 129; *ΔfimA*, flask: 60; *ΔfimA*, tube: 53. These ancestral polymorphisms primarily presented in clusters or within intergenic loci with frequencies <10%, consistent with our definition of these “polymorphisms” as likely sequencing or mutation calling noise—either due to difficulty mapping in non-unique genomic regions or non-randomness in tagmentation during library prep (see dataset at https://github.com/BehringerLab/FimA_Experimental_Evolution).

### Statistical analysis of genomic data

To identify parallel evolution within and among evolutionary treatments, we focused on nonsynonymous SNPs. Here, we constructed a matrix of every gene containing a non-synonymous SNP in at least one population and counted the number of SNPs in that gene for each population. Using this matrix, we assessed mean genetic distance and conducted an analysis of similarity within and among treatments using the Bray–Curtis method with the vegan package in R (Oksanen et al., 2025). Using the same matrix, we then performed principle component analysis to assess between population similarity and identify characteristic mutations associated with each treatment using the PCA function from the FactoMineR package in R (Lê et al., 2008).

## Supplementary Material

qraf048_Supplemental_Files

## Data Availability

Genomic sequencing data generated for analysis in this study is deposited on NCBI’s Sequencing Read Archive, BioProject number: PRJNA1297787. Code generated for statistical analysis and data visualization is deposited on the Behringer Lab’s GitHub repository at: https://github.com/BehringerLab/FimA_Experimental_Evolution.
