# Supplementary material for: Historical contingency limits adaptive diversification in a spatially structured environment
Source: Evol Lett. 2025 Dec 16;10(1):118–34. doi: 10.1093/evlett/qraf048 (PMC12870851; doi:10.1093/evlett/qraf048)
Supplement: qraf048_Supplemental_Files [file qraf048_supplemental_files.zip › Biofilm_Paper_Evol_Letters_SupplementalText.docx]

**Supplemental Figures and Tables**

Figure S1: Biofilm production after 48h culture at 25°C.

Figure S2: Pre-transfer culture density throughout experimental evolution after 48h culture at 25°C.

Figure S3: Fitness assessment following 48h co-culture at 25°C.

Figure S4: Genomic location of *arcA* mutations enriched in flask populations.

Figure S5: Early nucleotide-level parallelism in experimental evolution datasets.

Table S1: All mutations over 10% frequency for each population

Table S2: Early parallelism in the LTEE

**Figure S1: Biofilm production after 48h culture at 25°C.** Red dashed line indicates the mean value for the WT ancestor, blue dashed line indicates the mean value for the *ΔfimA* ancestor. Error bars represent 95% confidence interval of the mean.

**
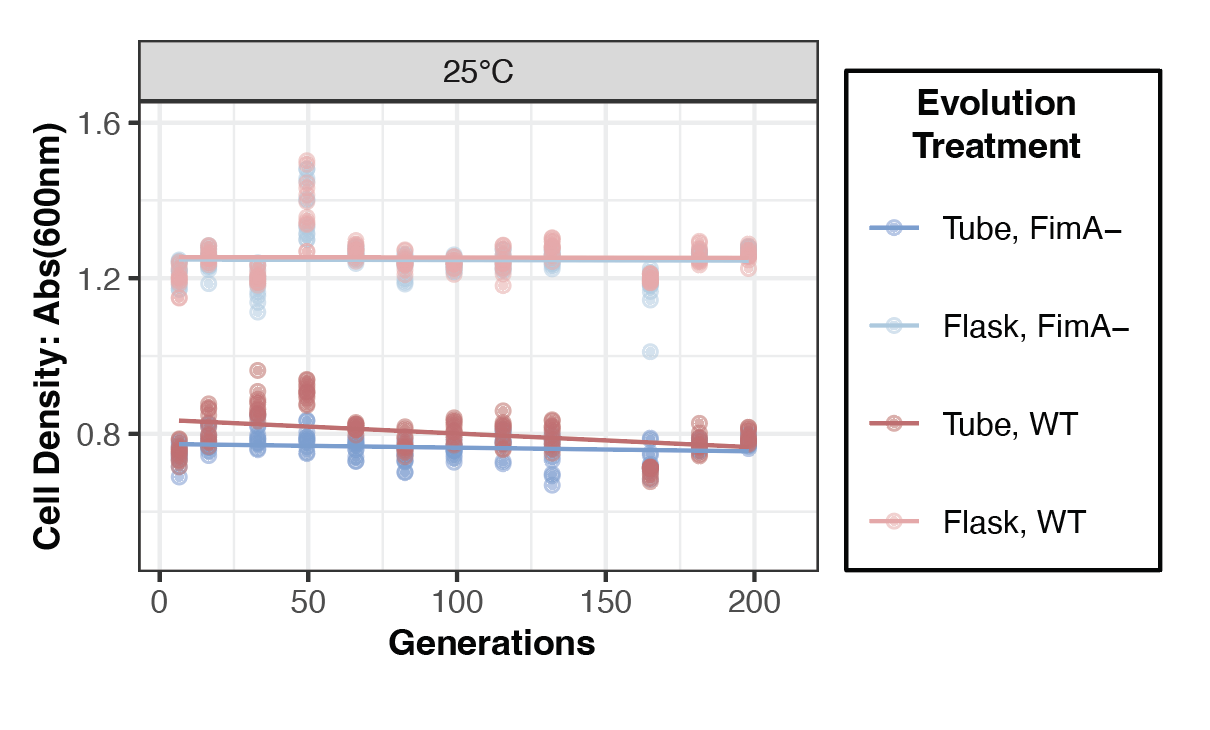
**

**Figure S2: Pre-transfer culture density throughout experimental evolution after 48h culture at 25°C.** Lines indicate a linear fit of the change in density over time.

**
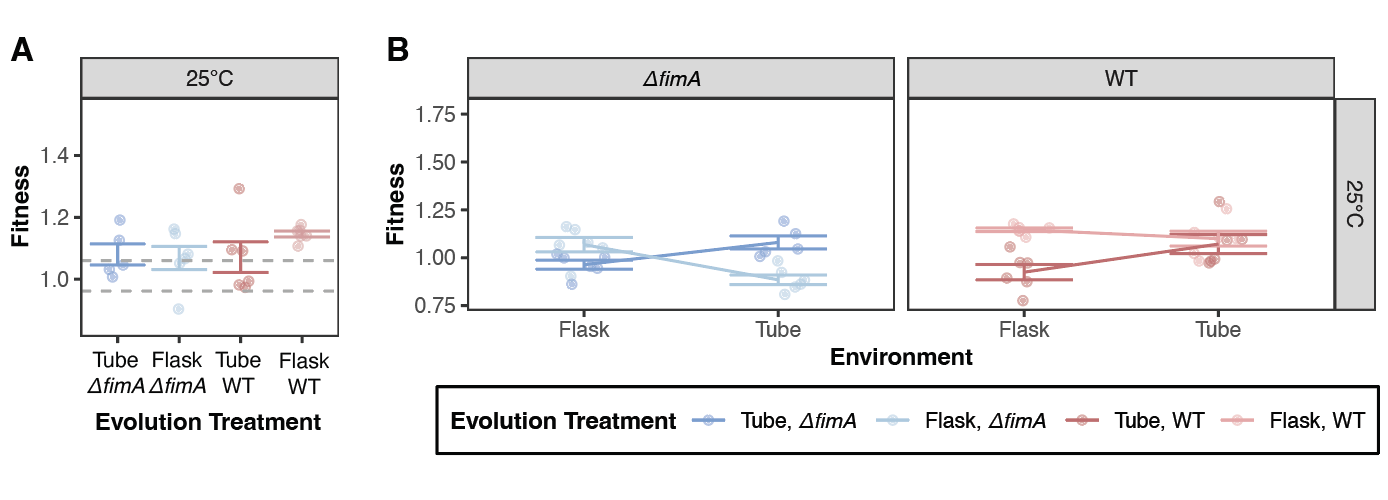
**

**Figure S3: Fitness assessment following 48h co-culture at 25°C.** Fitness is assessed by competitive co-culture in evolved and reciprocal environments. (A) Fitness of each population in their evolved environment after 91 days relative to the ancestor strain of the same genetic background (e.g. evolved *ΔfimA*-tube with *ΔfimA* ancestor). (B) Fitness of each population in their evolved or reciprocal environment relative to the ancestor strain of the same genetic background. Lines illustrate fitness relationships by connecting the mean fitness for each evolved genotype-environment combination, error bars represent 95% confidence intervals of the mean.


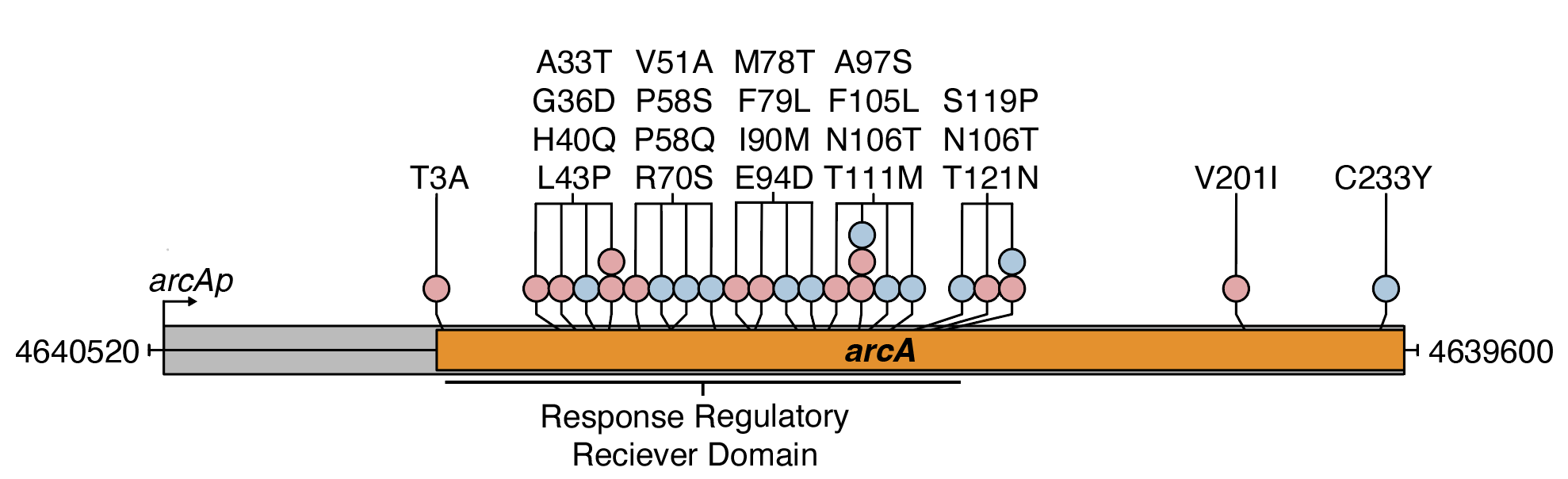


**Figure S4: Genomic location of *arcA* mutations enriched in flask populations.** Genic region of *arcA* locus shown, highlighting mutations observed in this experiment indicated above the labeled genes. Shapes indicate mutation types: SNPs, circles; colors indicate evolutionary treatment: red, WT-flask; blue, *ΔfimA*-flask.

**Figure S5: Early nucleotide-level parallelism in experimental evolution datasets.** A) Parallel polymorphisms across the first 2000 generations of evolution in the LTEE. Gray points and lines indicate unique polymorphisms, red points and lines indicate polymorphisms shared across two or more populations. B) Parallel polymorphisms in this experiment. Grey bars indicate unique polymorphisms, red bars indicate polymorphisms shared within an evolution treatment, blue bars indicate polymorphisms shared across multiple evolution treatments.
