# Supplementary figures and images for: Historical contingency limits adaptive diversification in a spatially structured environment
Source: Evol Lett. 2025 Dec 16;10(1):118–34. doi: 10.1093/evlett/qraf048 (PMC12870851; doi:10.1093/evlett/qraf048)

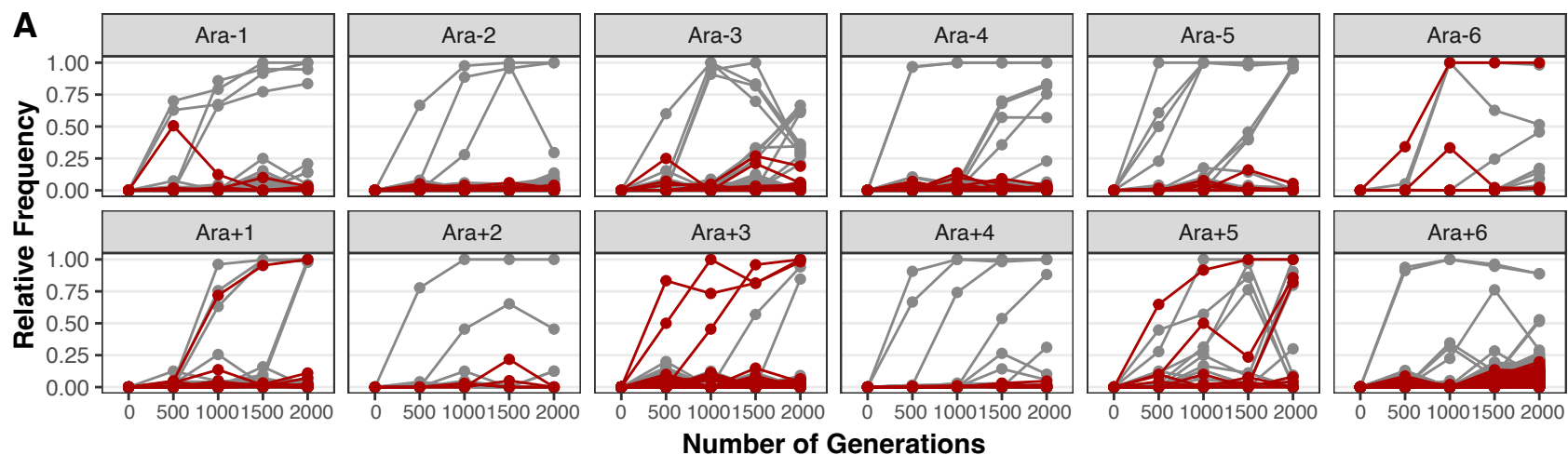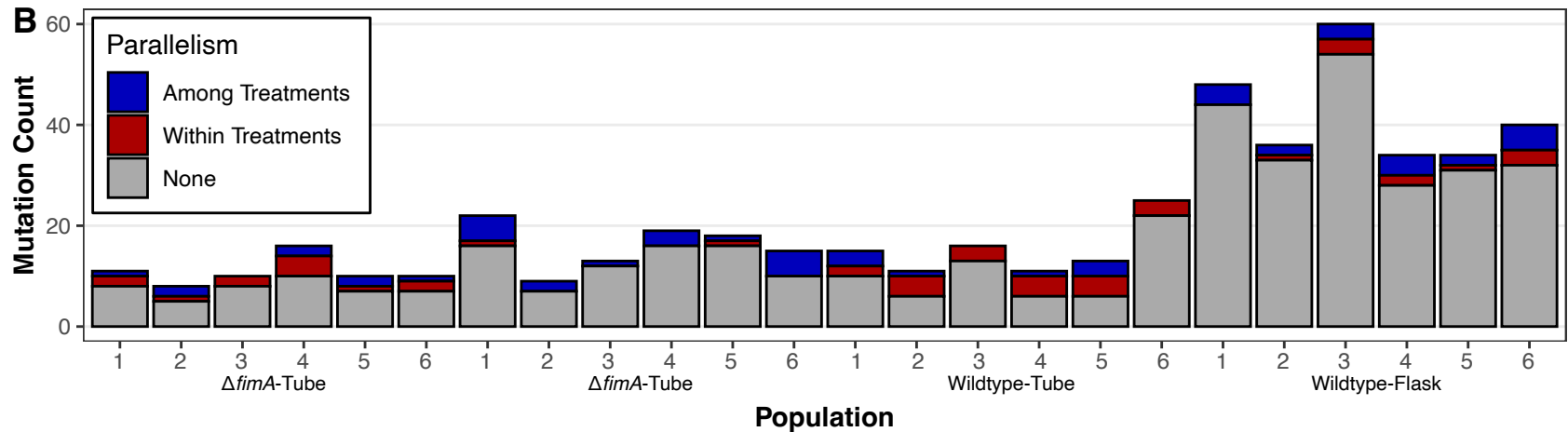

Supplement: qraf048_Supplemental_Files [file qraf048_supplemental_files.zip › FigureS5.pdf]

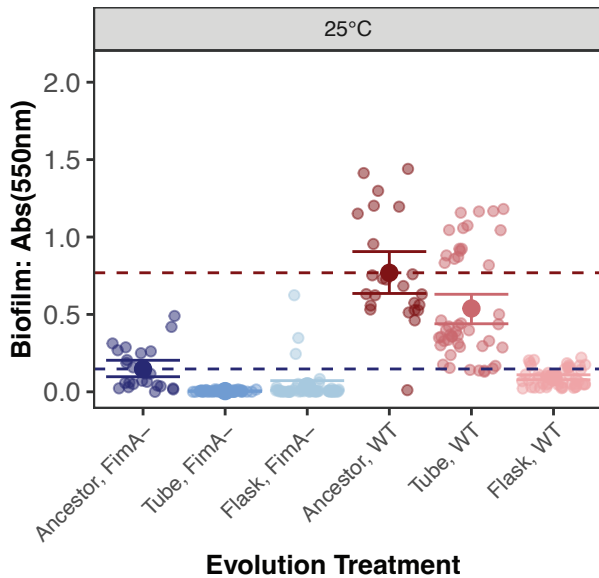

Supplement: qraf048_Supplemental_Files [file qraf048_supplemental_files.zip › Figure_S1_Biofilm_25.pdf]

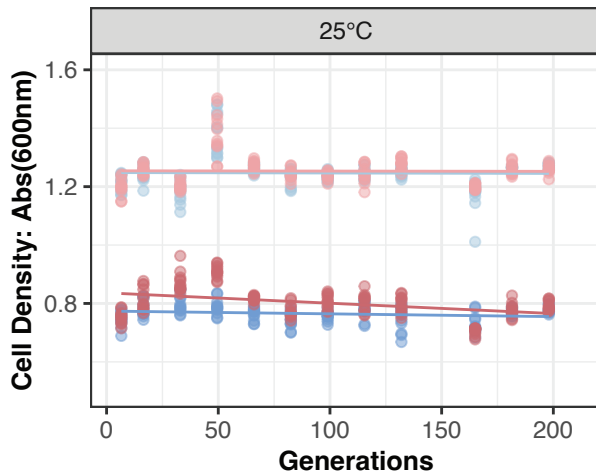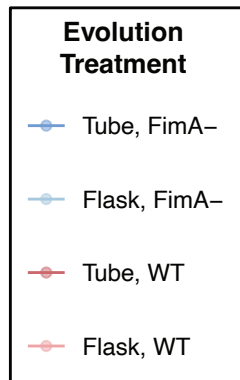

Supplement: qraf048_Supplemental_Files [file qraf048_supplemental_files.zip › Figure_S2_CultureDensity_25.pdf]

**A**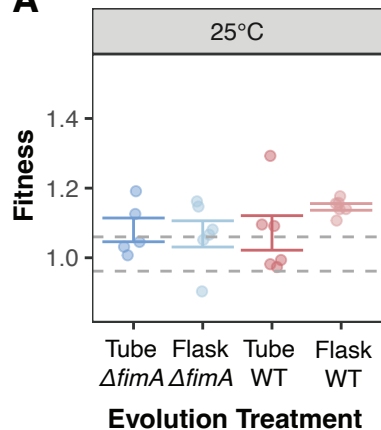**B**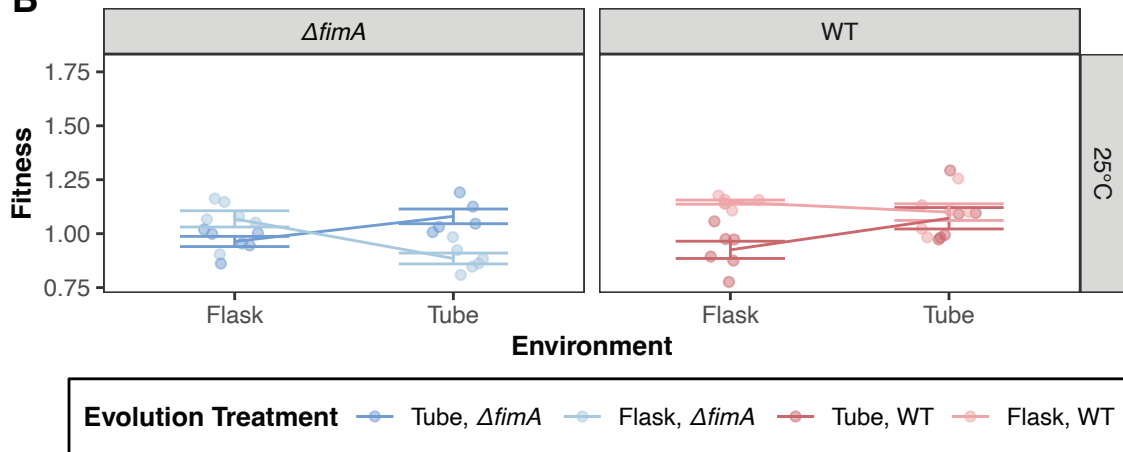

Supplement: qraf048_Supplemental_Files [file qraf048_supplemental_files.zip › Figure_S3.pdf]

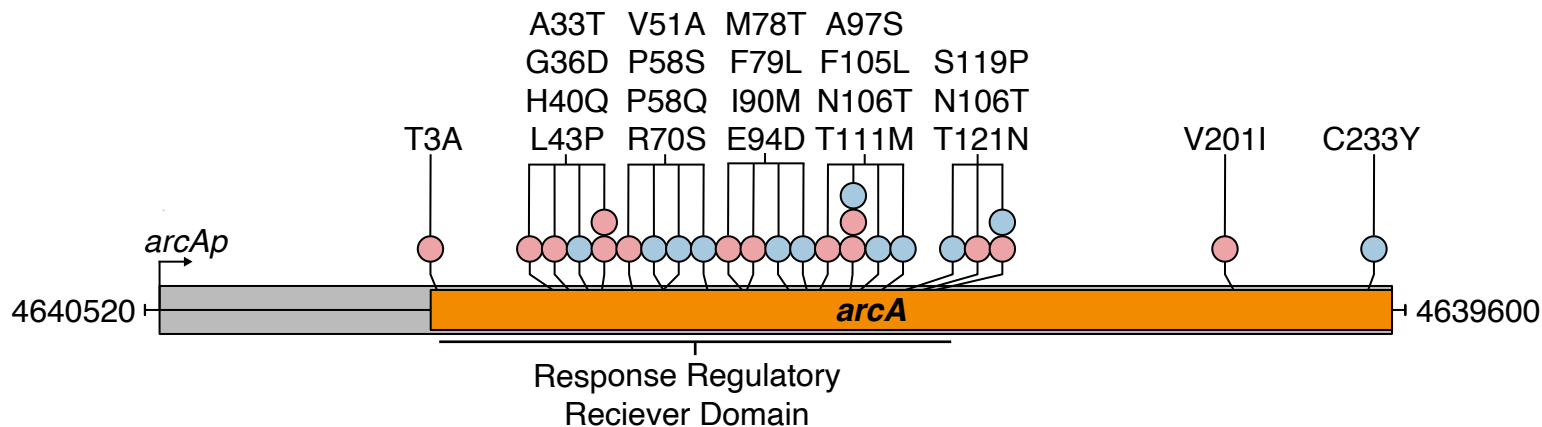

Supplement: qraf048_Supplemental_Files [file qraf048_supplemental_files.zip › Figure_S4.pdf]
